# Development and validation of an explainable machine learning model for mortality prediction in ICU patients with lung cancer

**DOI:** 10.3389/fcell.2026.1862263

**Published:** 2026-06-22

**Authors:** Jinhong Xia, Jingyuan Zhang, Siyu Zhang, Cheng Liu, Zhiyu Liu, Yuxi Zhao, Huaran Zhang, Min Shen

**Affiliations:** 1 Network Technology and Education Digitalization Center, Sichuan College of Traditional Chinese Medicine, Mianyang, China; 2 Key Laboratory of Non-coding RNA and Drug Discovery at Chengdu Medical College of Sichuan Province, School of Basic Medical Sciences, Chengdu Medical College, Chengdu, China; 3 Department of General Education, Sichuan College of Traditional Chinese Medicine, Mianyang, China; 4 The First Affiliated Hospital of Dalian Medical University, Dalian Medical University, Dalian, China; 5 School of Pharmacy, Chengdu Medical College, Chengdu, China; 6 Department of Basic Medical Sciences, Sichuan College of Traditional Chinese Medicine, Mianyang, China

**Keywords:** clinical prediction model, hospital mortality, intensive care unit, lung cancer, machine learning, MIMIC-IV, severity score, SHAP interpretability

## Abstract

**Background and Objective:**

Lung cancer is the leading cause of cancer-related death worldwide, with a particularly high mortality risk among patients admitted to the intensive care unit (ICU). Accurate and timely prediction of in-hospital mortality in this population is critical for clinical decision-making and resource allocation. However, existing severity scoring systems have demonstrated limited discriminative performance in this specific context. This study aimed to develop and validate an interpretable machine learning (ML) model for in-hospital mortality prediction in ICU patients with lung cancer using real-world clinical data from the MIMIC-IV(Medical Information Mart for Intensive Care IV) database.

**Materials and Methods:**

Retrospective cohort data were extracted from the MIMIC-IV database. A total of 1,120 ICU admissions of lung cancer patients were included (239 deaths, 21.3%). Candidate variables were systematically screened through univariate analysis, variance inflation factor (VIF) assessment, and bidirectional stepwise logistic regression—all performed exclusively on the training set (70%, n = 785) to prevent data leakage. Eight machine learning algorithms were developed: Logistic Regression (LR), Random Forest (RF), Neural Network (NN), Support Vector Machine (SVM), eXtreme Gradient Boosting (XGBoost), Gradient Boosting Machine (GBM), Adaptive Boosting (AdaBoost), and Decision Tree (DT). Model performance was evaluated on an independent test set (30%, n = 335) using six metrics—Brier score, calibration slope, F1 score, Youden index, area under the receiver operating characteristic curve (AUC), and accuracy (ACC)—with a composite scoring system to rank models. Interpretability was assessed using SHapley Additive exPlanations (SHAP). Clinical utility was evaluated through decision curve analysis (DCA) and comparison with traditional severity scores (Sequential Organ Failure Assessment (SOFA), Simplified Acute Physiology Score II (SAPS II), and Oxford Acute Severity of Illness Score (OASIS)). Subgroup and robustness analyses were also conducted.

**Results:**

Five variables were selected as final predictors: SOFA score, SAPS II score, OASIS score, Charlson Comorbidity Index (CCI), and minimum peripheral oxygen saturation (SpO_2_). The Logistic Regression model achieved the highest comprehensive score (42/48), with an AUC of 0.791 (95% CI: 0.731–0.850), Brier score of 0.137, F1 score of 0.550, Youden index of 0.482, accuracy of 0.746, and calibration slope of 0.801. The LR model significantly outperformed SOFA (AUC: 0.749, *P* = 0.034), SAPS II (AUC: 0.719, *P* = 0.011), and OASIS (AUC: 0.678, *P* < 0.001). SHAP analysis identified SOFA score as the dominant predictor, with SpO_2_contributing a negative influence. The model demonstrated stable and consistent performance across subgroups (mechanical ventilation, age, sex) and across 50 repeated random splits. DCA confirmed the model’s net clinical benefit at threshold probabilities of 0.10–0.70.

**Conclusion:**

We developed an explainable, LR-based mortality prediction model for ICU lung cancer patients, which outperforms traditional severity scoring systems and provides individual-level clinical interpretability through SHAP values. This model offers a practical and transparent tool to support critical care decision-making in this high-risk population.

## 1 Introduction

Lung cancer is the most prevalent and lethal malignancy worldwide, accounting for approximately 2.5 million new cases and 1.8 million deaths annually (Sung et al., 2021). Despite substantial advances in therapeutic strategies—including targeted therapy, immunotherapy, and stereotactic radiotherapy—the prognosis of lung cancer patients admitted to the ICU remains dismal (Soares et al., 2010; Zafra Poves et al., 2026). These patients frequently present with critical complications such as respiratory failure, sepsis, and multi-organ dysfunction, with reported in-hospital mortality rates ranging from 15% to 60% depending on disease severity and organ failure burden (Zhao et al., 2024; Sun et al., 2025).

Accurate early prediction of in-hospital mortality in ICU patients with lung cancer is of paramount clinical importance. Reliable prognostic tools can facilitate informed communication between clinicians and patients or families, guide triage decisions, optimize resource allocation in critically limited ICU settings, and potentially inform individualized treatment planning (Fleu et al., 2020; Nguyen et al., 2024). Currently, widely used severity scoring systems such as SOFA, SAPS II, and OASIS provide a broad assessment of critical illness severity; however, they were not specifically designed for oncological patients and exhibit limited discriminative ability in the lung cancer ICU context (Johnson et al., 2018; Sawicka et al., 2014; Cabrera Losada et al., 2024).

With the rapid growth of EHR infrastructure and widespread adoption of large clinical databases, machine learning (ML) techniques have emerged as powerful tools for building high-performance clinical prediction models (Huang et al., 2023; Bataill et al., 2021). ML algorithms can capture complex nonlinear interactions among clinical variables that traditional logistic regression or severity scores may miss. Several studies have explored ML-based mortality prediction in ICU patients with various cancer types, reporting superior performance compared to traditional severity scores (Gu and Lu, 2025; Peng et al., 2022; Wang et al., 2026). However, despite lung cancer being the most common cancer in ICU admissions, validated and interpretable ML models specifically developed for this population remain scarce (Zafra Poves et al., 2026; Sun et al., 2025; Li et al., 2025).

A critical barrier to clinical translation of ML models is their “black-box” nature, which limits clinician trust and adoption. (Rudin, 2019; El Arab and Al Moosa, 2025). Recent advances in explainable artificial intelligence, particularly SHAP, have provided a theoretically sound and model-agnostic framework for quantifying individual feature contributions to model predictions, thereby enhancing transparency and clinical interpretability (Luo et al., 2025; Nohara et al., 2022).This patient-level interpretability enables clinicians to understand risk drivers for individual ICU patients, support shared decision-making, guide timely interventions, and improve communication with patients and families—representing critical clinical value in real-world critical care practice.

**FIGURE 1:**
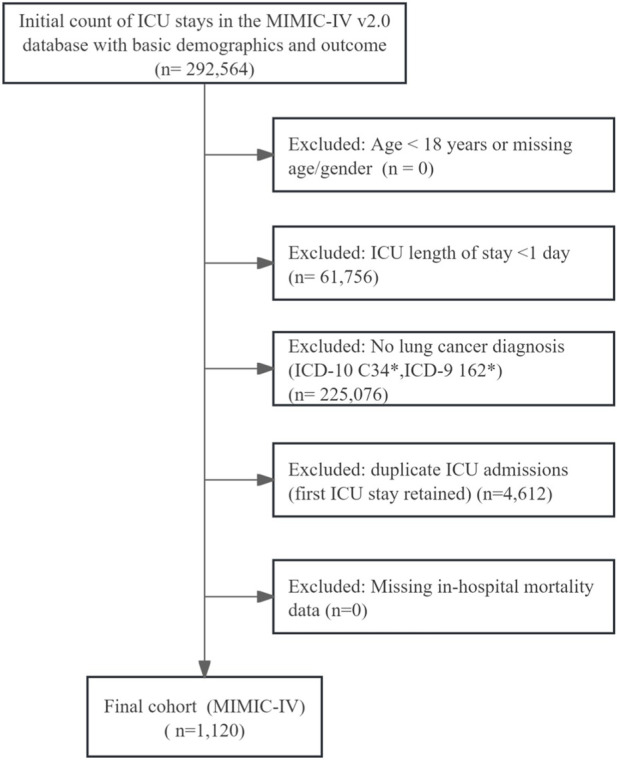
Study flow: cohort, split, modeling, and evaluation.

In this study, we leveraged the publicly available MIMIC-IV clinical database to develop and validate an explainable ML-based mortality prediction model for ICU patients with lung cancer. We compared eight ML algorithms using a rigorous composite scoring system incorporating six clinically relevant performance metrics. The best-performing model was further evaluated for clinical utility using DCA, compared against traditional severity scores, analyzed for interpretability using SHAP, and validated for subgroup robustness and stability. The research methodology is summarized in Figure 1.

## 2 Materials and methods

### 2.1 Data source

The data utilized in this study were derived from the Medical Information Mart for Intensive Care IV (MIMIC-IV, version 2.2) database, a publicly accessible critical care repository established and maintained by the Beth Israel Deaconess Medical Center (BIDMC) in Boston, Massachusetts (Johnson et al., 2023). MIMIC-IV contains de-identified clinical data from adult patients admitted to ICUs between 2008 and 2019, covering demographics, diagnoses, laboratory tests, vital signs, procedures, and outcomes. Access to the database was obtained following completion of the Collaborative Institutional Training Initiative (CITI) program. The present study relied solely on de-identified data from MIMIC-IV, whose use was approved by the institutional review boards of the Massachusetts Institute of Technology (Cambridge, MA, United States) and Beth Israel Deaconess Medical Center; informed consent requirements for the original data collection were waived.

### 2.2 Patient selection

Lung cancer patients admitted to the ICU were identified using International Classification of Diseases (ICD-9 and ICD-10) codes. Inclusion criteria were: (1) adult patients (age ≥ 18 years); (2) primary or secondary diagnosis of lung cancer; (3) ICU length of stay ≥ 24 h; and (4) complete records for key study variables. The sample enrollment process is illustrated in Figure 1. A total of 1,120 patients were included in the final analytic cohort.

### 2.3 Variable definition and preprocessing

Clinical variables were categorized as follows. Continuous variables included age, weight (kg), severity scores (SOFA, SAPS II, OASIS), vital sign extremes during the ICU stay (minimum mean arterial pressure [MAP], minimum SpO_2_, maximum respiratory rate, minimum/maximum heart rate, minimum/maximum systolic blood pressure, minimum/maximum diastolic blood pressure), and laboratory indices (minimum/maximum values for white blood cell count, hemoglobin, platelet, creatinine, blood urea nitrogen [BUN], sodium, potassium, bicarbonate, and glucose), as well as Charlson Comorbidity Index (CCI). Categorical variables included sex, race/ethnicity (White, Black/African American, Asian, Hispanic or Latino, Other/Unknown), insurance type (Medicare, Medicaid, Other), admission type (Emergency, Surgical Same Day Admission, Urgent, Observation, Elective), and binary indicators for mechanical ventilation, renal replacement therapy (RRT), vasopressor use, and comorbidities (hypertension, diabetes, heart failure, chronic kidney disease, COPD, coronary artery disease).

The primary outcome was in-hospital mortality (death_hospital: 0 = survived, 1 = died). Variables with missing rates below 20% were imputed using column-wise median imputation for continuous variables (Peng et al., 2026). Categorical variables were recoded as factors. The complete missing rate report is summarized in Supplementary Table S2.

### 2.4 Candidate variable selection

To mitigate the risk of information leakage from the test set, all variable selection procedures were restricted to the training set. Nineteen candidate variables of clinical relevance were thus included in the selection pipeline:

1. Univariate analysis: independent sample t-test for continuous variables; Fisher’s exact test for binary variables (2 × 2 tables) and chi-square test with Monte Carlo simulation (B = 10,000) for multi-category variables. Variables with *P* < 0.05 were retained.
2. Multicollinearity assessment: variance inflation factor (VIF) was calculated for all continuous variables using binary logistic regression. Variables with VIF > 10 were removed.
3. Bidirectional stepwise logistic regression (AIC criterion): the remaining significant variables were subjected to stepwise selection, yielding the final predictor set.

Results are reported in Supplementary Table S1.

### 2.5 Data splitting

The full cohort was randomly split into a training set (70%, n = 785) and an independent test set (30%, n = 335) using stratified random sampling based on the outcome variable (set.seed = 42), ensuring comparable mortality rates in both sets. All model training, hyperparameter tuning, and feature selection were performed exclusively on the training set. Final model evaluation was performed on the held-out test set.

### 2.6 Model evaluation and composite ranking

Eight machine learning algorithms were implemented in Python (version 3.10) using scikit-learn and XGBoost: logistic regression (L2, max_iter = 1,000), random forest (300 trees), neural network (64–32 units, ReLU, Adam, max_iter = 500), support vector machine (RBF kernel, probability enabled), XGBoost (300 trees, depth 4, learning rate 0.05), gradient boosting (300 trees, depth 3), AdaBoost (200 estimators), and decision tree (unpruned). Features were standardized with StandardScaler fitted on the training set only. The classification threshold for each model maximized the Youden index on the training set and was applied to the test set. All models used random_state = 42 for reproducibility.

Model performance on the test set was assessed using six metrics corresponding to the reference methodology:

**Table:** 

Metric	Direction
Brier score	Lower is better
Calibration slope	Closer to 1.0 is better
F1 score	Higher is better
Youden index	Higher is better
AUC	Higher is better
Accuracy (ACC)	Higher is better

A composite scoring system was applied to rank all eight models: for each metric, models were ranked from best to worst and assigned scores from 8 (best) to 1 (worst). For Brier score, ranking was ascending; for calibration slope, ranking was based on absolute deviation from 1.0; for the remaining four metrics, ranking was descending. The sum of scores across all six metrics yielded the total composite score (maximum = 48), with the highest-scoring model selected as the final recommended model.

### 2.7 Model interpretability (SHAP analysis)

SHAP values were computed for the best-performing model using the shap Python package (version 0.41). Three visualizations were generated: (A) a global beeswarm summary plot; (B) a waterfall plot for a representative high-risk patient; and (C) a bar chart of mean absolute SHAP values. Values for all test set patients were exported to shap_values_test.csv.

### 2.8 Comparison with traditional severity scores

The optimal machine learning (ML) model was benchmarked against three established intensive care unit (ICU) severity scoring systems—the Sequential Organ Failure Assessment (SOFA), the Simplified Acute Physiology Score II (SAPS II), and the Oxford Acute Severity of Illness Score (OASIS)—on the test set. Comparative performance metrics included the area under the receiver operating characteristic curve (AUC) with 95% confidence intervals (CIs) estimated via bootstrap with 1,000 iterations, the area under the precision-recall curve (AUPRC) with bootstrap-based 95% CIs, and the net reclassification improvement (NRI). Differences in AUC were assessed using DeLong’s method as implemented in the pROC R package. Cutoff values for the conventional severity scores were determined by maximizing the Youden index on the test set.

### 2.9 Decision curve analysis and calibration

DCA was performed to quantify the net clinical benefit of the best ML model relative to the “treat all” and “treat none” strategies across a range of threshold probabilities. Calibration was assessed by plotting the observed outcome rates against decile-based predicted probability groups and computing the calibration slope via logistic regression of the outcome on the logit of predicted probabilities (slope of 1.0 indicates perfect calibration).

### 2.10 Subgroup and robustness analyses

To evaluate model generalizability, subgroup analyses were performed for three pre-specified clinically relevant subgroups: mechanical ventilation status (Yes vs. No), age group (< 65 vs. ≥ 65 years), and sex (Male vs. Female). AUC with 95% bootstrap CI and Brier score were computed for each subgroup. Interaction effects between the model score and each subgroup variable were assessed using logistic regression with an interaction term.

Model stability was assessed by repeating the entire train-test split and retraining procedure 50 times with different random seeds, recording AUC, AUPRC, and Brier score distributions for all eight models.

### 2.11 Statistical analysis

All statistical analyses were performed using R (version 4.3.1) and Python (version 3.10). Continuous variables are presented as mean ± standard deviation (SD) or median (interquartile range [IQR]) as appropriate. Categorical variables are presented as counts and proportions. Comparisons between groups used independent t-tests or Mann-Whitney U tests for continuous variables and chi-square or Fisher’s exact test for categorical variables. A two-sided *P* < 0.05 was considered statistically significant. All figures were generated using the ggplot2 R package and the matplotlib Python library.

Sample size and power consideration: The study included 1,120 ICU admissions with 239 events (21.3% mortality), meeting the standard requirement of ≥100 events and ≥10 events per predictor for clinical prediction model development and validation. This sample size ensured sufficient statistical power for model training, internal validation, and stable performance estimation.

## 3 Results

### 3.1 Study population and baseline characteristics

A total of 1,120 ICU admissions of lung cancer patients from the MIMIC-IV database were included in the analysis. The study cohort comprised 239 in-hospital deaths (21.3%) and 881 survivors (78.7%). After stratified random splitting, 785 patients (70.1%) were assigned to the training set and 335 patients (29.9%) to the test set. The study flowchart is shown in Figure 1.

Baseline characteristics of the full cohort, stratified by outcome (Survived vs. Died) and by dataset split (Training vs. Test), are summarized in Table 1. Patients who died in hospital had significantly higher severity scores (SOFA, SAPS II, OASIS), higher CCI, higher rates of mechanical ventilation and vasopressor use, and lower SpO_2_values compared to survivors (all *P*<0.001). No significant differences in baseline characteristics were observed between the training and test sets (all *P* > 0.05), confirming appropriate stratified splitting.

**TABLE 1:** Basic characteristics of ICU lung cancer patients and comparison between the training and testing sets.

Variables	Overall (n = 1120)	Survived (n = 881)	Died (n = 239)	P	Training set (n = 785)	Test set (n = 335)	P
Age, years	69.12 ± 10.81	68.76 ± 10.83	70.44 ± 10.64	0.033	69.22 ± 11.11	68.88 ± 10.10	0.632
Sex, n (%)	​	​	​	0.220	​	​	0.229
Female	544 (48.6)	419 (47.6)	125 (52.3)	​	391 (49.8)	153 (45.7)	​
Male	576 (51.4)	462 (52.4)	114 (47.7)	​	394 (50.2)	182 (54.3)	​
Race/ethnicity, n (%)	​	​	​	0.138	​	​	0.751
White	802 (71.6)	653 (74.1)	149 (62.3)	​	551 (70.2)	251 (74.9)	​
Black/African american	104 (9.3)	78 (8.9)	26 (10.9)	​	75 (9.6)	29 (8.7)	​
Asian	—	—	—	​	—	—	​
Hispanic or latino	23 (2.1)	16 (1.8)	7 (2.9)	​	19 (2.4)	4 (1.2)	​
Other/Unknown	129 (11.5)	86 (9.8)	43 (18.0)	​	96 (12.2)	33 (9.9)	​
Insurance, n (%)	​	​	​	0.138	​	​	0.499
Medicare	529 (47.2)	404 (45.9)	125 (52.3)	​	379 (48.3)	150 (44.8)	​
Medicaid	68 (6.1)	52 (5.9)	16 (6.7)	​	45 (5.7)	23 (6.9)	​
Other	523 (46.7)	425 (48.2)	98 (41.0)	​	361 (46.0)	162 (48.4)	​
Admission type, n (%)	​	​	​	<0.001	​	​	0.498
Emergency (EW EMER.)	576 (51.4)	435 (49.4)	141 (59.0)	​	399 (50.8)	177 (52.8)	​
Observation admit	135 (12.1)	104 (11.8)	31 (13.0)	​	102 (13.0)	33 (9.9)	​
Surgical same-day admission	154 (13.8)	148 (16.8)	6 (2.5)	​	104 (13.2)	50 (14.9)	​
Elective	31 (2.8)	30 (3.4)	1 (0.4)	​	25 (3.2)	6 (1.8)	​
Other	34 (3.0)	24 (2.7)	10 (4.2)	​	23 (2.9)	11 (3.3)	​
Charlson comorbidity Index	4.46 ± 2.96	4.24 ± 2.98	5.26 ± 2.75	<0.001	4.50 ± 2.94	4.37 ± 3.02	0.503
SOFA score	4.39 ± 3.20	3.70 ± 2.63	6.93 ± 3.80	<0.001	4.35 ± 3.24	4.47 ± 3.12	0.571
SAPS II score	39.77 ± 12.25	37.65 ± 10.77	47.59 ± 14.10	<0.001	40.06 ± 12.44	39.08 ± 11.78	0.221
OASIS score	32.00 ± 8.08	30.54 ± 7.34	37.41 ± 8.40	<0.001	32.08 ± 8.22	31.82 ± 7.76	0.629
Mechanical ventilation, n (%)	​	​	​	<0.001	​	​	0.231
No	667 (59.6)	578 (65.6)	89 (37.2)	​	477 (60.8)	190 (56.7)	​
Yes	453 (40.4)	303 (34.4)	150 (62.8)	​	308 (39.2)	145 (43.3)	​
Vasopressor use, n (%)	​	​	​	<0.001	​	​	0.168
No	796 (71.1)	672 (76.3)	124 (51.9)	​	568 (72.4)	228 (68.1)	​
Yes	324 (28.9)	209 (23.7)	115 (48.1)	​	217 (27.6)	107 (31.9)	​
SpO_2_ minimum, %	90.32 ± 6.00	90.88 ± 5.43	88.25 ± 7.40	<0.001	90.23 ± 5.64	90.51 ± 6.78	0.474

Continuous variables are presented as mean ± SD; categorical variables as n (%). *P*-values are derived from t-test or Mann–Whitney U test for continuous variables and chi-square or Fisher’s exact test for categorical variables. P_outcome compares Survived vs. Died groups; P_split compares Training vs. Test sets.

### 3.2 Variable selection

Among the 19 candidate clinical variables entered into the selection pipeline (performed on the training set only), 12 were significantly associated with in-hospital mortality in univariate analysis (*P* < 0.05). Multicollinearity assessment confirmed that no variable exceeded VIF > 10. Following bidirectional stepwise logistic regression (AIC criterion), five variables were selected as the final predictors: SOFA score, SAPS II score, OASIS score, Charlson Comorbidity Index (CCI), and minimum SpO_2_during the ICU stay. Full univariate and stepwise regression results are presented in Supplementary Table S1.

The inclusion of SOFA, SAPS II, and OASIS was intentionally retained because these three scores capture distinct physiological dimensions of critical illness. SOFA mainly reflects acute organ dysfunction, SAPS II integrates both acute and chronic physiological parameters, while OASIS emphasizes early physiological status within the first hour of ICU admission. Their complementary information improves model robustness without severe multicollinearity, as supported by VIF values.

Notably, although mechanical ventilation and vasopressor use were highly significant in univariate analysis (*P* < 0.001), they were not retained in the final model after stepwise regression, likely due to their collinearity with severity scores. SOFA score demonstrated the strongest univariate association (t-test *P* = 4.73 × 10^-21^).

### 3.3 Model performance comparison and composite ranking

**FIGURE 2:**
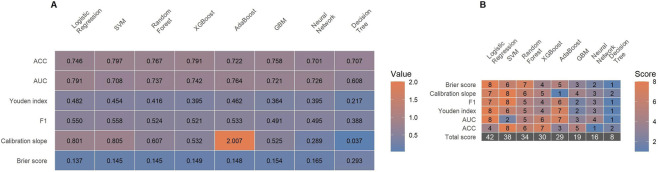
Test-set performance: **(A)** six metrics; **(B)** composite scores (max 48).

All eight ML models were trained on the training set and evaluated on the independent test set. Detailed performance metrics for each model are presented in Table 1 of Figure 2 (tabular summary embedded in Figure 2A), with the composite ranking shown in Figure 2B.

The Logistic Regression (LR) model achieved the highest composite score of 42/48, emerging as the best-performing model overall. It ranked first in Brier score (0.137) and Youden index (0.482), and placed highly in AUC (0.791) and calibration slope (0.801). Although other top-ranked models such as SVM showed comparable predictive performance, the logistic regression model offers superior clinical interpretability and bedside practicality without complex computational requirements, making it more feasible for routine clinical use in the ICU.The full composite scoring breakdown is presented in Table 2 below:

**TABLE 2:** Comprehensive scoring of eight machine learning models (testing set).

Model	AUC	Brier	F1	Youden	ACC	Cal. Slope	Total score
**Logistic Regression**	**0.791**	**0.137**	**0.550**	**0.482**	0.746	0.801	**42**
SVM	0.708	0.145	0.558	0.454	**0.797**	0.805	38
Random forest	0.737	0.145	0.524	0.416	0.767	0.607	34
XGBoost	0.742	0.149	0.521	0.395	0.791	0.532	30
AdaBoost	0.764	0.148	0.533	0.462	0.722	2.007	29
GBM	0.721	0.154	0.491	0.364	0.758	0.525	19
Neural network	0.726	0.165	0.495	0.395	0.701	0.289	16
Decision tree	0.608	0.293	0.388	0.217	0.707	0.037	8

Cal. Slope: Calibration slope; Total Score: composite score (max = 48). Bold values indicate the highest composite score among all models, corresponding to the best overall performance.

### 3.4 Performance curves of the best model

**FIGURE 3:**
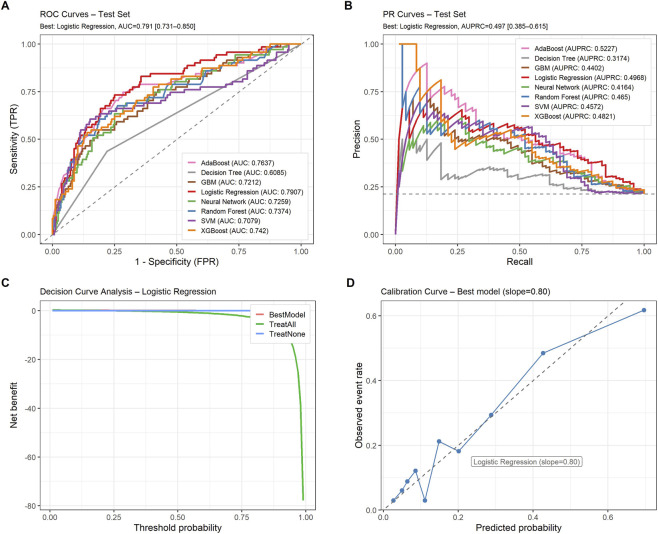
Best-model test-set curves: **(A)** ROC; **(B)** PR; **(C)** DCA; **(D)** calibration.

The performance of the best LR model on the test set was further characterized through four complementary visualizations (Figure 3):

ROC curve (Figure 3A): The LR model achieved an AUC of 0.791 (95% CI: 0.731–0.850) on the test set, with DeLong test comparisons confirming statistically significant superiority over SVM (*P* = 0.039), GBM (*P* = 0.012), Neural Network (*P* = 0.008), and Decision Tree (*P* < 0.001). At the optimal Youden-index threshold (0.198), sensitivity was 73.2% and specificity was 75.0%.

Precision-Recall curve (Figure 3B): The area under the precision-recall curve (AUPRC) for the LR model was 0.497 (95% CI: 0.385–0.615), substantially exceeding the baseline prevalence of 21.3%.

Decision Curve Analysis (Figure 3C): DCA demonstrated that the LR model provided positive net clinical benefit over both “treat all” and “treat none” reference strategies across threshold probabilities ranging from approximately 0.10 to 0.70, indicating clinical utility across a wide range of risk thresholds.

Calibration Curve (Figure 3D): The calibration curve showed that predicted probabilities from the LR model closely tracked observed event rates across deciles, with a calibration slope of 0.801 (ideally 1.0), indicating mild under-dispersion but overall acceptable calibration.

### 3.5 Comparison with traditional severity scores

The LR model was systematically compared with three traditional ICU severity scores—SOFA, SAPS II, and OASIS—on the test set. Results are summarized in Table 3.

**TABLE 3:** Logistic Regression vs. traditional severity scores.

Metric	LR model	SOFA	SAPS II	OASIS
Cutoff (Youden)	0.198	0.344	0.306	0.573
AUC (95% CI)	0.791 (0.731–0.850)	0.749 (0.681–0.817)	0.719 (0.653–0.785)	0.678 (0.601–0.754)
*P* vs. LR (DeLong)	—	0.034	0.011	< 0.001
AUPRC (95% CI)	0.497 (0.385–0.615)	0.466 (0.359–0.588)	0.454 (0.338–0.571)	0.414 (0.299–0.529)
NRI (total)	Reference	0.086	0.160	0.143
NRI (event)	Reference	0.113	−0.014	0.169
NRI (nonevent)	Reference	−0.027	0.174	−0.027

LR: logistic regression; SOFA: sequential organ failure assessment; SAPS II: Simplified Acute Physiology Score II; OASIS: oxford acute severity of illness score; NRI: Net Reclassification Improvement (continuous, using ML, model as reference). P-values from DeLong test.

The LR model significantly outperformed all three severity scores in discriminative ability. The AUC of the LR model (0.791) was significantly higher than that of SOFA (0.749, *P* = 0.034), SAPS II (0.719, *P* = 0.011), and OASIS (0.678, *P* < 0.001). Similarly, the LR model achieved higher AUPRC values than all three comparators. The NRI analyses further confirmed the reclassification improvement of the ML model over traditional scores.

### 3.6 SHAP interpretability analysis

**FIGURE 4:**
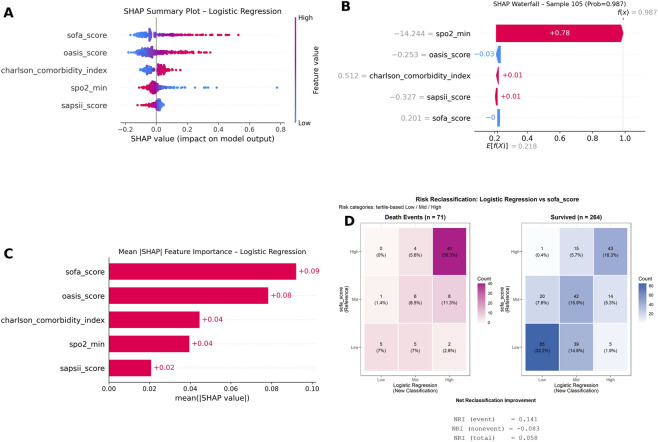
**(A–C)** SHAP summary, waterfall, and mean |SHAP|; **(D)** LR vs SOFA reclassification (NRI).

SHAP analysis was conducted on the best LR model to provide both global and individual-level explanations of model predictions (Figure 4).

The global SHAP summary plot (Figure 4A) revealed that SOFA score was the dominant predictor of in-hospital mortality, with higher SOFA scores substantially increasing predicted mortality risk. OASIS score and SAPS II score also showed strong positive associations. CCI contributed positively, while minimum SpO_2_ exhibited a negative SHAP contribution (lower SpO_2_ values increased mortality risk). These SHAP-derived insights are clinically coherent and consistent with established critical care knowledge.

Higher SOFA, OASIS, and SAPS II scores indicate more severe organ dysfunction and acute physiological deterioration, thereby increasing mortality risk. A higher Charlson Comorbidity Index (CCI) reflects greater chronic disease burden, which further elevates mortality risk. In contrast, a higher minimum SpO_2_ represents better oxygenation and respiratory function, which is associated with lower in-hospital mortality. These directional effects are clinically consistent and pathophysiologically meaningful for critically ill lung cancer patients.

The waterfall plot for an exemplary high-risk patient (Figure 4B) demonstrated how a high SOFA score (+contribution), elevated OASIS score (+contribution), and low SpO_2_ (−contribution) collectively pushed the predicted probability substantially above the baseline. The mean |SHAP| importance bar chart (Figure 4C) confirmed the rank ordering: SOFA > OASIS > SAPS II > CCI > SpO_2_.

To complement these SHAP-based explanations, we further visualized how the LR model reclassified patients compared with SOFA using Net Reclassification Improvement (NRI) analysis. Figure 4D illustrates the risk reclassification matrix (tertile-based Low/Mid/High) for the LR model versus SOFA score, separately for death events and survivors, with NRI (event), NRI (nonevent), and NRI (total) annotated. This visual representation supports the numerical NRI results reported in Table 3.

### 3.7 Subgroup analysis

**FIGURE 5:**
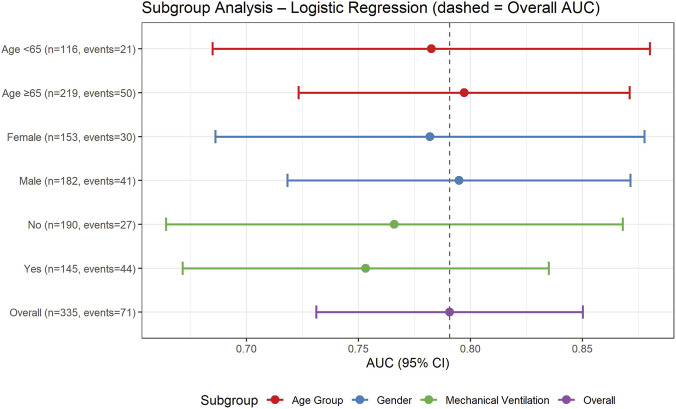
Subgroup AUC (95% CI) vs overall test AUC.

To assess the robustness and generalizability of the LR model across clinically relevant patient subgroups, we performed stratified AUC analyses for three pre-specified subgroups (Figure 5 and Table 4).

**TABLE 4:** Subgroup AUC of the Logistic Regression model on the testing set.

Subgroup	n	Events	AUC	95% CI	Brier
Overall	335	71	0.791	0.731–0.850	0.137
Mechanical ventilation					
No	190	27	0.766	0.664–0.868	0.100
Yes	145	44	0.753	0.671–0.835	0.186
Age group					
< 65 years	116	21	0.782	0.685–0.880	0.138
≥ 65 years	219	50	0.797	0.723–0.871	0.137
Sex					
Male	182	41	0.795	0.718–0.871	0.144
Female	153	30	0.782	0.686–0.878	0.129

Across all six subgroups, the AUC of the LR model remained consistently in the range of 0.753–0.797, with overlapping confidence intervals and no evidence of significant model × subgroup interaction (all interaction *P* > 0.05). These findings indicate that the model’s predictive performance is stable and does not vary substantially with mechanical ventilation status, age, or sex.

### 3.8 Model stability (50 repeated random splits)

**FIGURE 6:**
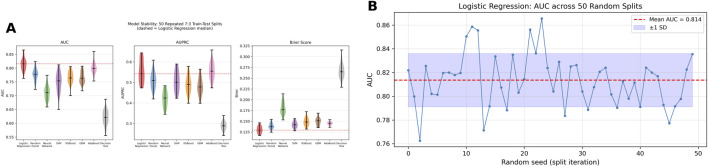
**(A)** 50-split distributions; **(B)** LR AUC over seeds (mean ±1 SD).

To confirm that model performance was not dependent on a particular random partition, we repeated the entire training–testing split 50 times with different random seeds and retrained all eight models (Figure 6).

Across 50 random splits, the LR model exhibited the most stable performance: mean AUC = 0.814 ± 0.023, mean Brier = 0.137 ± 0.013. The consistently low standard deviation (coefficient of variation < 3%) confirmed that the model’s performance reported on the primary split was representative and reproducible. The LR model ranked first in mean AUC across all 50 repetitions, further validating its selection as the optimal model.

## 4 Discussion

In this study, we developed and validated an explainable machine learning model for predicting in-hospital mortality in ICU patients with lung cancer using real-world data from MIMIC-IV. Compared with recent machine learning studies for mortality prediction in oncologic ICU patients, our work provides clearer novelty and clinical value. We used a rigorous composite evaluation system to screen eight algorithms and confirmed that logistic regression has more stable performance and better clinical interpretability than complex black-box models. Meanwhile, strict training-set-only variable selection and SHAP individual explanation enhance the transparency and practicability of the model, which fills the gap of clinically translatable prediction tools specifically for ICU lung cancer patients (Huang et al., 2023; Chris et al., 2019; Ledziński et al., 2025).

The main findings are as follows: (1) among eight candidate ML algorithms, the Logistic Regression model achieved the highest composite performance score; (2) five clinical variables—SOFA score, SAPS II score, OASIS score, CCI, and minimum SpO_2_—were identified as the optimal predictor set through a rigorous, training-set-only variable selection procedure; (3) the LR model significantly outperformed conventional severity scoring systems in discriminative ability; (4) SHAP analysis provided transparent, clinically coherent individual-level and global explanations of model predictions; and (5) subgroup and repeated-split analyses confirmed the model’s stability and generalizability.

The development of reliable prognostic tools for oncological patients in the ICU has been an area of active investigation (Soares et al., 2010; Sun et al., 2025). Critically ill lung cancer patients represent a particularly challenging subgroup, given the interplay between cancer-related immunosuppression, treatment-related organ toxicity, and acute illness severity (Zafra Poves et al., 2026; Zhao et al., 2024). Our cohort included 1,120 patients with a mortality rate of 21.3%, consistent with prior reports from cancer-specific ICU studies (Nguyen et al., 2024; Sawicka et al., 2014). The observed mortality was notably lower than that in some earlier series, possibly reflecting improvements in oncological supportive care over the 2008–2019 MIMIC-IV period, as well as advances in ICU management (Johnson et al., 2023).

Our variable selection process—confined strictly to the training set—identified five final predictors: SOFA, SAPS II, OASIS, CCI, and minimum SpO_2_. The co-selection of all three severity scores might superficially appear to introduce redundancy; however, each score captures distinct dimensions of physiological derangement and organ dysfunction. SOFA primarily reflects acute organ failure; SAPS II integrate more chronic physiological parameters; OASIS emphasizes physiological data collected within the first hour of ICU admission (Johnson et al., 2018). Their complementary inclusion is further supported by VIF values all well below 10, indicating acceptable collinearity in the multivariable model. CCI captures underlying chronic disease burden, while minimum SpO_2_ directly quantifies hypoxemic severity—a cardinal feature of respiratory failure in lung cancer patients (Zhao et al., 2024; Cabrera Losada et al., 2024; Bataill et al., 2021).

Among eight ML algorithms evaluated using a rigorous six-metric composite scoring system, the Logistic Regression model ranked first (score 42/48, AUC 0.791). This finding may seem counterintuitive given the complexity of ensemble methods such as XGBoost or GBM, but is consistent with findings in other clinical prediction studies where simpler, well-calibrated models outperform complex ones in terms of overall clinical utility (Chris et al., 2019; Moons et al., 2019). Notably, the AdaBoost model—despite achieving a high AUC—received the worst calibration slope score (2.007) after applying the corrected scoring logic (|slope − 1| metric), substantially reducing its overall ranking. This underscores the importance of including calibration as a mandatory evaluation metric beyond discrimination, as a model with high AUC but poor calibration may be clinically misleading when used to estimate absolute probabilities for individual patients (Van Calster et al., 2019). The LR model demonstrated a calibration slope of 0.801, approaching the ideal value of 1.0, and good visual calibration on the decile-based calibration curve.

SHAP analysis provided mechanistically coherent interpretations of the LR model’s predictions. SOFA score emerged as the most influential predictor, consistent with its established role as the gold standard severity indicator in ICU patients (Sawicka et al., 2014; Ranzani et al., 2025). The positive SHAP contributions of severity scores and CCI, and the negative contribution of SpO_2_ (where low SpO_2_ → high mortality risk), align with fundamental pathophysiological principles of critical illness in lung cancer patients (Gu and Lu, 2025; Wang et al., 2026). Beyond providing global feature importance, SHAP waterfall plots enable the clinician to understand, for each individual patient, which specific clinical parameters are driving the elevated risk estimate—a capability that traditional severity scores entirely lack (Luo et al., 2025; Nohara et al., 2022). This individual-level explainability may facilitate clinical adoption, patient communication, and documentation.

The LR model demonstrated statistically significant superiority over all three traditional severity scores in AUC: vs. SOFA (*P* = 0.034), vs. SAPS II (*P* = 0.011), vs. OASIS (*P* < 0.001). NRI analyses further confirmed the reclassification improvement, with the most pronounced improvement observed versus SAPS II (NRI = 0.160). While all three severity scores are widely used in daily ICU practice, they share a fundamental limitation in oncological patients: they were derived from general ICU populations and do not specifically account for cancer-related prognostic factors such as comorbidity burden (CCI) or oxygenation deficits specific to lung cancer (Huang et al., 2023; Peng et al., 2022). The ML model, by integrating these additional dimensions and optimizing over the full joint predictor space, captures prognostic signal that severity scores alone cannot (Tang et al., 2025).

DCA confirmed that the LR model provides net clinical benefit over default management strategies across a wide range of threshold probabilities (0.10–0.70), indicating practical utility for clinical decision-making. This is particularly relevant in triage decisions regarding ICU admission, escalation of care, or transition to palliative management for lung cancer patients at high predicted mortality risk (Fleu et al., 2020; Rudin, 2019).

Subgroup analysis (Figure 6) demonstrated that AUC was remarkably consistent across mechanically ventilated vs. non-ventilated patients (0.753 vs. 0.766), younger vs. older patients (0.782 vs. 0.797), and male vs. female patients (0.795 vs. 0.782), with no significant interaction effects. These results indicate that the model does not suffer from differential performance across these clinically important subgroups—an important equity consideration for clinical deployment (El Arab and Al Moosa, 2025). Similarly, stability analysis across 50 repeated random splits (Figure 6) showed consistent mean AUC of 0.814 ± 0.023, confirming that the reported performance metrics are not an artifact of a favorable particular random split.

Several limitations of this study should be acknowledged. First, data were derived from a single center (BIDMC, Boston, United States), which may limit generalizability to other healthcare systems, geographies, or patient populations. Second, the variable selection identified only five predictors—including all three of the standard ICU severity scores—which may reflect the dominance of aggregate severity metrics and partial collinearity in the feature space. Additional cancer-specific variables (e.g., cancer stage, histological subtype, treatment history, performance status) were not available in MIMIC-IV in a complete or structured form, and their absence may have attenuated prognostic signals and led to slight underestimation of the model’s true predictive performance. Third, median imputation was applied for missing values due to multicollinearity issues with PMM-based multiple imputation; while missing rates were uniformly low, more sophisticated imputation strategies may be explored. Median imputation may slightly reduce feature variance and introduce mild underestimation of uncertainty, which could exert a minor influence on model stability and calibration. However, given the low missing rates (<20%) in our dataset and robust results across 50 repeated random splits, such potential effects were likely limited and did not materially alter the main conclusions.Fourth, the calibration slope of 0.801 indicated mild underestimation of mortality probability at high risk thresholds, which warrants cautious interpretation when using predicted probabilities for clinical decision-making. Future studies should focus on multi-center external validation and prospective clinical implementation to further improve model reliability and real-world applicability.

## 5 Conclusion

We developed and validated an explainable machine learning-based model for predicting in-hospital mortality in ICU patients with lung cancer using a large, real-world clinical database. The Logistic Regression model—incorporating five predictors (SOFA, SAPS II, OASIS, CCI, and minimum SpO_2_)—achieved superior discriminative and calibration performance compared to traditional severity scoring systems, with robust and consistent performance across patient subgroups and repeated validation experiments. SHAP analysis provided clinically coherent, patient-level interpretability. This model represents a transparent, practical, and validated tool that may augment clinical decision-making in the critical care management of lung cancer patients.

Accordingly, this model is particularly suitable for risk stratification, triage decision-making, early prognostic evaluation, and family communication for lung cancer patients upon ICU admission, supporting realistic clinical deployment in daily critical care practice.

## Data Availability

The data analyzed in this study were derived from the publicly available MIMIC-IV database (https://physionet.org/content/mimiciv/). Analytical scripts are available from the corresponding author upon reasonable request.
